# Temporal pattern of neuronal insulin release during *Caenorhabditis elegans* aging: Role of redox homeostasis

**DOI:** 10.1111/acel.12855

**Published:** 2018-11-19

**Authors:** Alicia N. Minniti, Héctor Arriagada, Soledad Zúñiga, Marcela Bravo‐Zehnder, Iván E. Alfaro, Rebeca Aldunate

**Affiliations:** ^1^ Departamento de Biología Celular y Molecular, Facultad de Ciencias Biológicas Pontificia Universidad Católica de Chile Santiago Chile; ^2^ Escuela de Biotecnología, Facultad de Ciencias Universidad Santo Tomás Santiago Chile; ^3^ Facultad de Ciencias, Centro de Biología Celular y Biomedicina Universidad San Sebastián Santiago Chile; ^4^ Departamento de Biología, Facultad de Ciencias Naturales y Exactas Universidad de Playa Ancha Valparaíso Chile; ^5^ Fundación Ciencia & Vida Santiago Chile

**Keywords:** aging, *Caenorhabditis elegans*, neuronal insulin, redox homeostasis

## Abstract

The insulin‐IGF‐1/DAF‐2 pathway has a central role in the determination of aging and longevity in *Caenorhabditis elegans* and other organisms. In this paper, we measured neuronal insulin secretion (using INS‐22::Venus) during *C. elegans* lifespan and monitored how this secretion is modified by redox homeostasis. We showed that INS‐22::Venus secretion fluctuates during the organism lifetime reaching maximum levels in the active reproductive stage. We also demonstrate that long‐lived *daf‐2* insulin receptor mutants show remarkable low levels of INS‐22::Venus secretion. In contrast, we found that short‐lived mutant worms that lack the oxidation repair enzyme MSRA‐1 show increased levels of INS‐22::Venus secretion, specifically during the reproductive stage. MSRA‐1 is a target of the insulin‐IGF‐1/DAF‐2 pathway, and the expression of this antioxidant enzyme exclusively in the nervous system rescues the mutant insulin release phenotype and longevity. The *msra‐1* mutant phenotype can also be reverted by antioxidant treatment during the active reproductive stage. We showed for the first time that there is a pattern of neuronal insulin release with a noticeable increment during the peak of reproduction. Our results suggest that redox homeostasis can modulate longevity through the regulation of insulin secretion, and that the insulin‐IGF‐1/DAF‐2 pathway could be regulated, at least in part, by a feedback loop. These findings highlight the importance of timing for therapeutic interventions aimed at improving health span.

## 1 INTRODUCTION

Aging research in *Caenorhabditis elegans* and other organisms has shown that lifespan is genetically and environmentally determined. Loss of function mutations in the insulin‐IGF‐1/DAF‐2 pathway can dramatically increase lifespan in *C. elegans* (Kenyon, Chang, Gensch, Rudner, & Tabtiang, 1993) and in other animal models (Holzenberger et al., 2003). The increase in longevity is dependent on the forkhead transcription factor DAF‐16, an ortholog of the human FOXO3a transcription factor (Ogg et al., 1997), and this pathway is conserved from nematodes to mammals (Hesp, Smant, & Kammenga, 2015; Martins, Lithgow, & Link, 2016). DAF‐16 extends lifespan by upregulating genes involved in cellular stress‐response, antimicrobial response, and metabolism, as well as by downregulating life‐shortening genes (Murphy, 2006; Murphy et al., 2003). Among the numerous genes upregulated by DAF‐16, we find those implicated in decreasing ROS levels, such as superoxide dismutases, glutathion S transferases (Honda & Honda, 1999; Murphy, 2006), and methionine sulfoxide reductase (MSRA‐1) (Minniti et al., 2009).

The *C. elegans* genome encodes a single insulin/IGF‐1‐like receptor (DAF‐2); however, it carries several genes that encode for insulin‐like peptides (ILPs). Forty members of the insulin family have been found through genetic and bioinformatic analyses (Li & Kim, 2008), and several ILPs were shown to regulate longevity and developmental processes (Fernandes de Abreu et al., 2014). Many ILPs show neuronal expression or are expressed in specific subsets of neurons, while a few are expressed in the intestine. Both types of peptides regulate longevity through DAF‐16 (Li & Kim, 2008; Murphy, Lee, & Kenyon, 2007). Evidence gathered in *C. elegans* indicates that DAF‐16 influences lifespan cell non‐autonomously by regulating the insulin pathway in several tissues (Libina, Berman, & Kenyon, 2003). The strongest evidence showing the role of this pathway in neurons comes from experiments in which the expression of the DAF‐2 receptor exclusively in the nervous system is sufficient to abolish lifespan extension of *daf‐2* mutants (Dillin, Crawford, & Kenyon, 2002; Wolkow, Kimura, Lee, & Ruvkun, 2000). In mammals, there is also evidence of the importance of the insulin/IGF signaling in the central nervous system and its relationship with aging (Broughton & Partridge, 2009). *Irs2* (insulin receptor substrate 2) knockout mice are diabetic and as a consequence they have a shorter lifespan (Selman, Partridge, & Withers, 2011); however, if the deletion of the *Irs2* gene is brain‐specific, the mice are long lived even though they have a diabetic phenotype (Taguchi, Wartschow, & White, 2007).

It is possible that insulin from the nervous system is not only transcriptionally regulated (Berendzen et al., 2016; Libina et al., 2003; Murphy et al., 2007) but it may also be controlled at the secretion level throughout the animal's life. Does insulin release from neurons remain constant during *C. elegans* lifespan or does it change over time? Does the pattern of insulin release influence the aging process?

Current research has focused on identifying genes that regulate secretion of insulin/IGFs from neurons in *C. elegans*. However, there are no studies on whether there is a temporal course of insulin release during *C. elegans* lifespan. Some genes have been described to increase insulin release when mutated, such as *goa‐1* (a subunit of a trimeric G protein) and *tom‐1* (a syntaxin binding protein). Interestingly, these mutants also show a reduction in the animals lifespan (Ch'ng, Sieburth, & Kaplan, 2008). Others, such as *gon‐1*, decrease the release of some insulin peptides (*ins‐7* and *daf‐28*) (Yoshina & Mitani, 2015). Consequently, mutations that reduce insulin secretion cause an increase in the animal's lifespan (Yoshina & Mitani, 2015), while mutations that exacerbate insulin secretion decrease lifespan (Ch'ng et al., 2008).

Given the importance of the insulin‐IGF‐1/DAF‐2 pathway in the determination of lifespan and the aging process across species, we focused our studies on characterizing neuronal insulin secretion during *C. elegans* lifetime to find out whether there is a specific temporal pattern of secretion. We also analyzed if this pattern could be modulated by the insulin‐IGF‐1/DAF‐2 pathway itself.

Additionally, we evaluated if any of the DAF‐16 target genes could modulate the pathway at the level of insulin secretion from neurons. As mentioned before, DAF‐16 induces in part the expression of the cell´s antioxidant machinery (Murphy, 2006; Sun, Chen, & Wang, 2017). Our previous work shows that the DAF‐16 upregulated target MSRA‐1, an oxidation repair enzyme ortholog of the *Drosophila* and human MsrA genes, is necessary to maintain wild‐type (Wt) lifespan (Lee et al., 2005; Minniti et al., 2009). Unlike other antioxidant enzymes such as SOD‐1 and SOD‐3 (Doonan et al., 2008; Van Raamsdonk & Hekimi, 2012), the absence of the single *C. elegans* MsrA gene *(msra‐1)* causes a 30% decrease in lifespan (Minniti et al., 2009). This function is conserved from yeast to rodents (Chung et al., 2010; Koc, Gasch, Rutherford, Kim, & Gladyshev, 2004; Moskovitz et al., 2001). We also showed that MSRA‐1 is expressed in the nervous system of Wt and *daf‐2* mutants (Minniti et al., 2009). In *Drosophila,* this oxidation repair enzyme also activates FOXO (DAF‐16) increasing its nuclear localization (Chung et al., 2010). However, the mechanism involved is unknown.

In this paper, we show that there is a consistent pattern of INS‐22::Venus secretion levels from neurons during the animal´s lifespan, and that the levels of secretion show a noticeable increase during the active reproductive stage (ARS: days 1–3 of adulthood). Long‐lived *daf‐2* insulin receptor mutants show the same age dependent pattern; however, the levels of INS‐22::Venus secretion are extremely low, and are only detectable during the ARS. In contrast, in the short‐lived *msra‐1* mutant, INS‐22::Venus secretion increases significantly only during ARS. Expression of *msra‐1* exclusively in the nervous system rescues the Wt pattern of neuronal INS‐22::Venus release and longevity. The *msra‐1* mutant phenotype can also be reversed by antioxidant treatment only during the ARS. Our results suggest that redox homeostasis can modulate longevity through the regulation of insulin secretion and that the insulin‐IGF‐1/DAF‐2 pathway might be regulated at least in part by a feedback loop at the level of insulin secretion that involves DAF‐16 targets.

## 2 RESULTS

### 2.1 The rate of neuronal INS‐22::Venus release changes during Wt *C. elegans* lifespan

**Figure 1:**
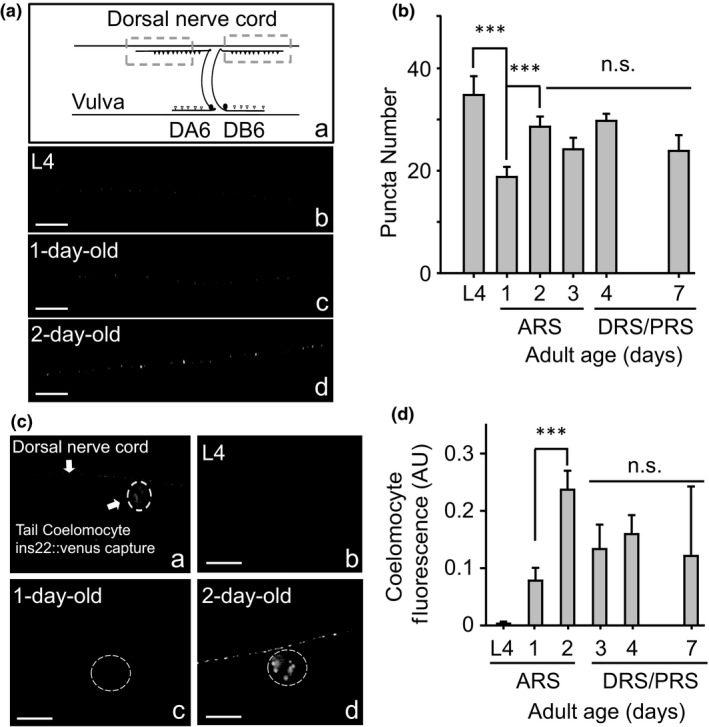
Neuronal insulin release increases during the young adult stage in Wt *Caenorhabditis elegans* ***.*** Panel A shows puncta fluorescence of INS‐22::Venus in dorsal axons. Aa shows the area analyzed in the worms. Ab, c, and d show representative images of INS‐22::Venus in dorsal axons at different stages. B. Quantification of puncta at different ages including L4, the adult active reproductive stage (ARS), and the declining and post‐reproductive stages (DRS/PRS). C. Secreted INS‐22::Venus accumulates in coelomocytes. Ca. The image highlights one of the two posterior coelomocytes used for our analyses. The thick arrow shows the dorsal axon. Cb, c, and d show representative images of INS‐22::Venus captured by coelomocytes at different stages. D. The graph shows fluorescence of INS‐22::Venus accumulated in the coelomocytes at different stages. Scale bar, 5 μm. Data are means ± *SE* from at least three independent assays. Student's *t* test was used for statistical analysis. ***p < 0.0001. At least 30 animals were tested at each time point

To investigate whether there are changes in the pattern of insulin release during the different *C. elegans* adult stages, we used a transgenic strain that expresses INS‐22 fused to Venus (under the control of the *unc‐129* promoter) in nine DA and DB cholinergic neurons (Ch'ng et al., 2008). This in vivo secretion assay allows us to discriminate between neuropeptide expression and its release from neurons since its synthesis is maintained constant during lifespan due to the promoter selected. INS‐22 is one of many *C. elegans* neuropeptides, and it is endogenously expressed in most neurons, including DA and DB neurons (Baugh, Kurhanewicz, & Sternberg, 2011; Li & Kim, 2008). We estimated the secretion of INS‐22::Venus by comparing the fluorescence present in neurons: Venus fluorescence in axonal processes (Diagram in Figure 1Aa) vs. secreted insulin: Venus fluorescence accumulated in the coelomocytes (Figure 1Ca). We expect a negative correlation between puncta number and coelomocyte fluorescence. However, there could be a time delay between secretion and coelomocyte uptake.

First, we analyzed neuronal puncta in DA6 and DB6 neurons. As expected, we can detect INS‐22::Venus in the axons of these neurons (Figure 1A). We analyzed fluorescence images from adult individuals: 1‐, 2‐, and 3‐day‐old worms (we consider this period as the active reproductive stage: ARS), 4‐day‐old worms (declining reproductive stage: DRS), and 7‐day‐old worms (post‐reproductive stage: PRS) (Figure 1A,B). In order to compare larval with adult INS‐22::Venus secretion, we also evaluated the L4 stage (Figure 1). In our model, we found that the neurons are producing INS‐22::Venus in all the developmental stages analyzed, including the L4 stage. Even though there is a decrease in the number of fluorescent puncta from the L4 stage to day 1 of adulthood, by day 2, the number of puncta is equivalent to that of the L4 (Figure 1b).

On the other hand, there is a significant increase in INS‐22::Venus secretion measured as accumulation of fluorescence in the coelomocytes (Figure 1Cb–d) from the L4 stage (where insulin is barely detectable) through all adult stages analyzed. Figure 1D shows that INS‐22::Venus secretion increases in the ARS reaching its maximum level in adult day 2. In DRS and PRS, INS‐22::Venus is maintained at similar levels as in day 1.

In order to explore if this pattern of INS‐22::Venus secretion is specific for this peptide or a common behavior of other neuropeptides, we evaluated two other neuropeptides: ANF::GFP and NLP‐21::Venus. We found a similar temporal secretion pattern for these two peptides (Supporting Information Figure S1).

### 2.2 Downregulation of the insulin pathway through the DAF‐2 receptor affects INS‐22::Venus release from neurons

Next, we decided to investigate whether mutations in *daf‐2* could affect INS‐22::Venus release.

**Figure 2:**
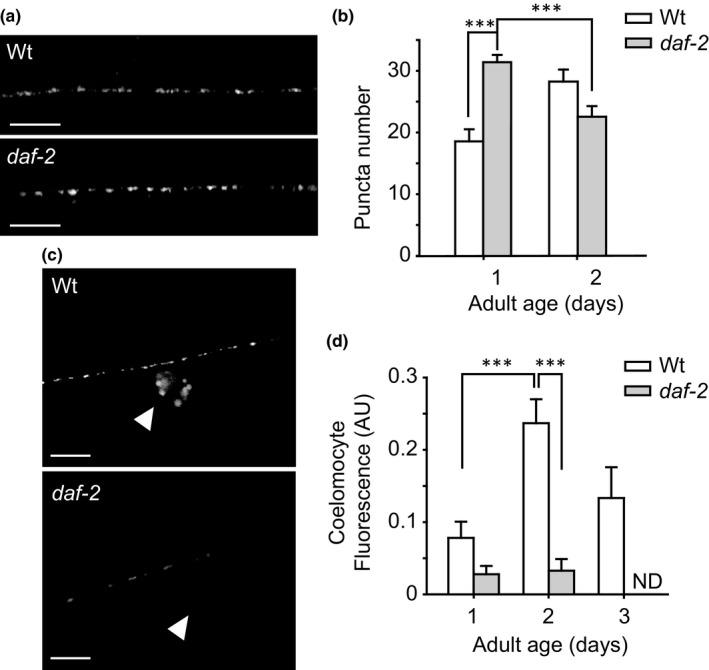
*daf‐2* mutants show decreased levels of INS‐22::Venus release. (a) Representative images of INS‐22::Venus in dorsal axons of Wt and *daf‐2* worms. (b) The graph shows the quantification of puncta number in Wt and *daf‐2* worms. (c) Secreted INS‐22::Venus in the coelomocytes (arrowheads) in Wt and *daf‐2* worms. (d) The graph shows the quantification of fluorescence of INS‐22::Venus accumulated in the coelomocytes of Wt and *daf‐2* worms. ND: not detectable. Scale bar, 5 μm. Data are means ± *SE* from at least three independent assays. Student's *t* test was used for statistical analysis. ***p < 0.0001. At least 20 animals were tested at each time point. *daf‐2* worms were grown at 16°C until they reached the L4 stage and then shifted to 23°C

Figure 2 shows that neurons in *daf‐2* mutants have increased number of puncta in day 1 of adulthood compared to the Wt. When we measured secretion (coelomocyte fluorescence), we could barely detect INS‐22::Venus in the coelomocytes of 1‐ and 2‐day‐old adult worms (Figure 2c,d). As in the Wt, we observed that there is a peak of secretion during the ARS albeit hardly detectable in this mutant.

### 2.3 The absence of MSRA‐1 increases INS‐22::Venus release during the active reproductive stage

Current evidence shows that not all the components that influence the organism redox homeostasis have the same consequences in the determination of lifespan in *C. elegans* (Doonan et al., 2008). When the insulin pathway is downregulated, (*daf‐2* mutants) the antioxidant machinery is activated and longevity is extended.

We previously showed that the insulin‐IGF‐1/DAF‐2 pathway regulates the antioxidant enzyme MSRA‐1 in *C. elegans* and the deletion of the *msra‐1* gene shortens the worms’ lifespan (Minniti et al., 2009). Supporting Information Figure S2 shows that by day 5 of adulthood *msra‐1* mutants have increased levels of proteins that are oxidized in methionines. There are other antioxidant enzymes controlled by this pathway such as the Mn‐superoxide dismutase (Honda & Honda, 1999). However, they do not cause a decrease in lifespan when absent (Doonan et al., 2008). This evidence along with our results related to INS‐22::Venus secretion in *daf‐2* mutants (Figure 2) led us to speculate that MSRA‐1 might influence aging and lifespan not only by maintaining redox balance in the animal´s tissues but also by controlling the levels of insulin secretion during lifespan.

**Figure 3:**
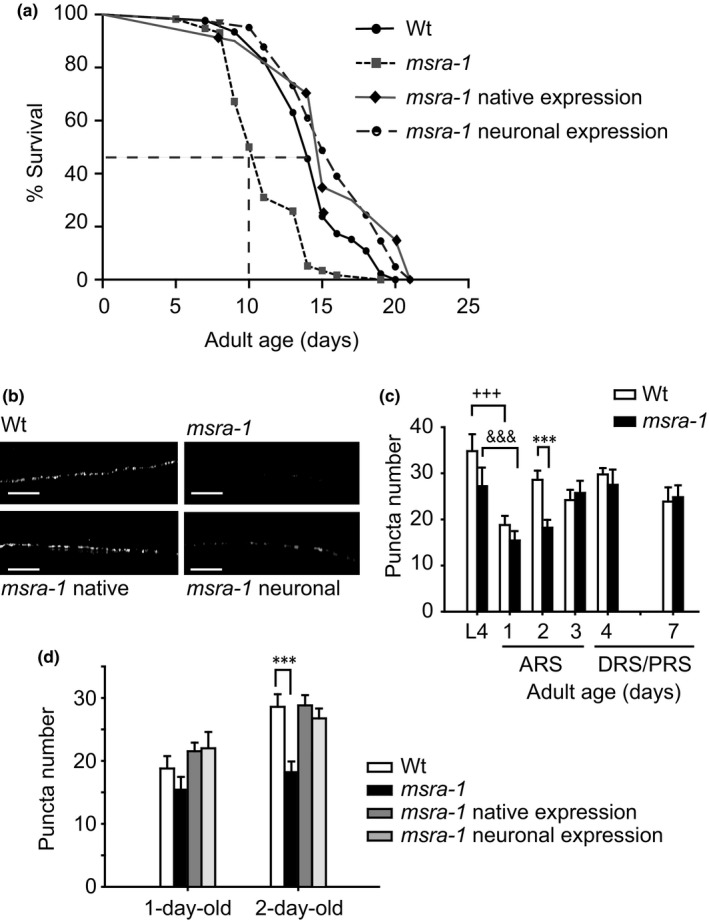
*msra‐1* mutants show fewer INS‐22::Venus puncta in the dorsal nerve cord than Wt during the ARS. (a) Survival curves of Wt, *msra‐1* and rescued *msra‐1* worms. *p* Value ≤ 0.0001 for comparison between Wt and *msra*‐*1(tm1421)* and *msra*‐*1(tm1421)* with rescued strains. Total number of animals tested was at least 90 per strain in three independent assays. (b) The representative photographs show puncta number of INS‐22::Venus in dorsal axons of 2‐day‐old Wt, *msra‐1* mutants, and rescued *msra‐1* mutant worms. (c) Puncta quntification in Wt and *msra‐1* mutants at different ages. (d) The graph shows puncta number in *msra‐1* rescued strains. Scale bar, 5 μm. Data are means ± *SE* from at least three independent assays. Student's *t* test was used for statistical analysis. ***p < 0.0001 when comparing Wt with *msra‐1* mutants. +++*p* < 0.0001, when comparing Wt L4 with Wt 1‐day‐old. ^&&&^ *p* < 0.0001, when comparing L4 *msra‐1* mutants with 1‐day‐old *msra‐1* mutants. At least 25 animals per strain were tested for each time point

Therefore, we investigated INS‐22::Venus secretion from neurons in the absence of MSRA‐1. Even though we demonstrated that MSRA‐1 is expressed in most tissues including neurons (Minniti et al., 2009), we wanted to test whether its expression exclusively in neurons was necessary and sufficient to rescue Wt longevity in *msra‐1* mutants. Figure 3a shows that this is indeed the case. Considering that by day 10 of adulthood 50% of *msra‐1* mutant worms are already dead, all further assays were performed up to day 7 to avoid selecting for the fittest worms.

Figure 3b–d shows the presence of INS‐22::Venus in neurons of WT and *msra‐1* L4 larvae and adult worms. We found that in *msra‐1* mutants INS‐22::Venus decreases (puncta number) with respect to the Wt (Figure 3b,c). This decrease is more pronounced in day 2 of adulthood while in L4 larvae and day‐1 adults there is no detectable difference. The difference in day 2 is abolished when we express *msra‐1* under the control of its native promoter (expression in most tissues) or when we express *msra‐1* exclusively in neurons (Figure 3d).

Next, we evaluated if the decrease in neuronal INS‐22::Venus during the active reproductive stage in *msra‐1* mutants correlates with increased INS‐22::Venus secretion seen as accumulation of INS‐22::Venus in coelomocytes.

**Figure 4:**
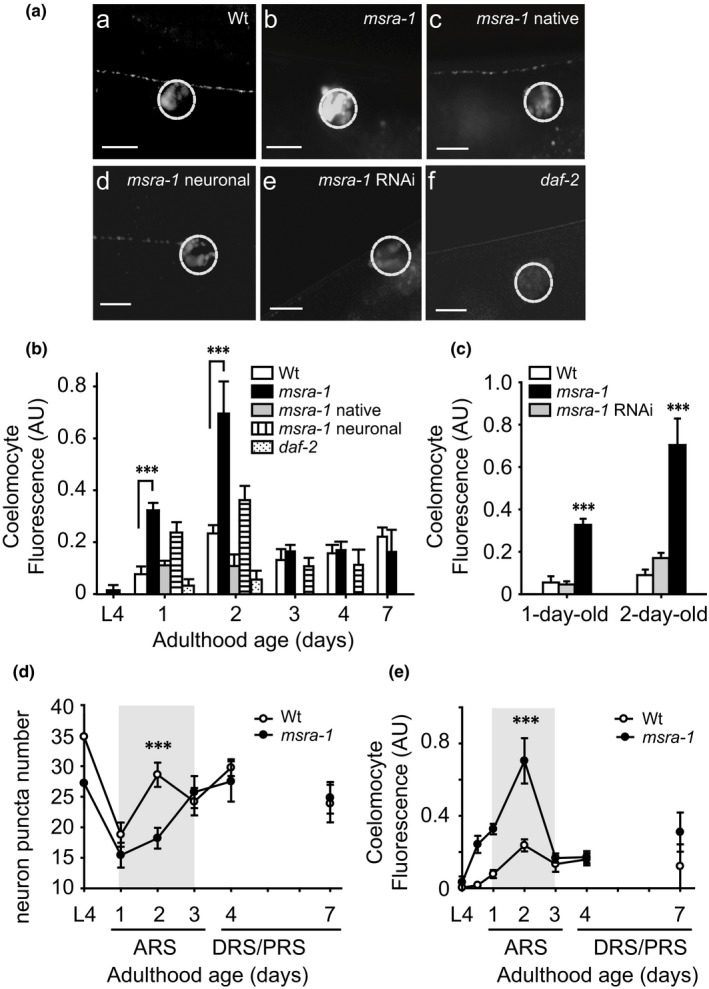
*msra‐1* mutants show increased insulin release during the ARS. A. Insulin released from motorneurons is captured by the coelomocytes in 2‐day‐old worms. B. Quantification of fluorescence of INS‐22::Venus accumulated in the coelomocytes of worms of the same genotypes shown in A at different stages. C. The graph shows that *msra‐1* RNAi knockdown in all tissues except in neurons maintains INS‐22::Venus accumulation in coelomocytes. The graphs in D and E show the comparison of puncta number and INS‐22::Venus coelomocyte fluorescence during the ARS and DRS/PRS between Wt and *msra‐1* mutants. Scale bar, 5 μm. Data are means ± *SE* from at least three independent assays. Student's *t* test was used for statistical analysis. ***p < 0.0001. At least 25 animals were tested per strain at each time point

Figure 4 shows INS‐22::Venus accumulation in coelomocytes of 2‐day‐old adult Wt (Figure 4Aa) and *msra‐1* mutants (Figure 4Ab). The graph in Figure 4B shows the quantification of INS‐22::Venus accumulation in the L4 stage and several adult stages that include the ARS, the DRS, and the PRS. The pattern of temporal secretion is similar in Wt and *msra‐1* mutants, but there is a clear increase of INS‐22::Venus release only during the ARS. By day 3, secretion is equivalent to that of the Wt (Figure 4B). Figure 4Ac,B shows that Wt levels of secretion are rescued when *msra‐1* is expressed under the control of its native promoter (expression in most tissues (Minniti et al., 2009)) or exclusively in neurons (Figure 4Ad,B), supporting the role of MSRA‐1 in INS‐22::Venus secretion from neurons.

Using an RNAi strategy, we further tested the hypothesis that *msra‐1* is particularly relevant in neurons in terms of INS‐22::Venus secretion. In this experimental approach, we used a Wt background strain, which is resistant to RNAi in neurons. We have already shown that using this strategy MSRA‐1 expression is maintained only in neurons (Minniti et al., 2015). Figure 4 Ae,C shows that the downregulation of *msra‐1* in all tissues except in the nervous system maintains INS‐22::Venus release at Wt levels. For comparison purposes, we included INS‐22::Venus accumulation levels in coelomocytes of *daf‐2* mutants in Figure 4 Af,B. We then compared the patterns of INS‐22::Venus release in Wt and *msra‐1* mutants from the L4 stage to day 7 of adulthood. Figure 4D,E shows that while INS‐22::Venus decreases in neurons of *msra‐1* mutants (Figure 4D), the coelomocytes of these mutants show an significant increase in INS‐22::Venus accumulation (Figure 4E). This phenomenon is restricted to days 1 and 2 of the active reproductive stage. By day 3 of adulthood, there is no difference in INS‐22::Venus levels between Wt and mutant worms in neurons or in coelomocytes. Supporting Information Figure S1b,c shows that the absence of MSRA‐1 does not increase secretion of the other neuropeptides tested (ANF::GFP and NLP‐21::Venus).

In order to test the possibility that the clear accumulation of INS‐22::Venus in the coelomocytes of *msra‐1* mutants is due to an increase in its expression and/or to exacerbated endocytosis in the coelomocytes, we performed the following control experiments. First, we analyzed INS‐22::Venus protein expression in 2‐day‐old Wt, *msra‐1* and *daf‐2* worms by Western blot (Supporting Information Figure S3a,b). This approach allowed us to detect and compare only the insulin pro‐peptide fused to Venus. However, we were not able to analyze the secreted form using this technique because Venus and GFP (used as co‐transformation marker in our strains) are of similar size. The data show that the synthesis of this pro‐peptide is fairly equivalent in all strains since the differences are not statistically significant.

Subsequently, we tested the hypothesis that *msra‐1* mutant coelomocytes present exacerbated endocytosis. We used a strain that expresses a secreted form of GFP in muscle cells (strain GS1912, (Fares & Greenwald, 2001)). We compared GFP accumulated in the coelomocytes of hermaphrodites of this strain (Wt background) with GFP accumulated in the coelomocytes of *msra‐1* mutants. We did not find increased accumulation of secreted GFP in *msra‐1* mutant coelomocytes (Supporting Information Figure S3c,d). Therefore, the accumulation of INS‐22:Venus in the coelomocytes of *msra‐1* worms is neither due to increased expression nor to exacerbated endocytosis in these cells.

An unexpected result is that the absence of MSRA‐1 (*msra‐1; daf‐2* double mutant) does not seem to significantly affect INS‐22::Venus secretion levels in the *daf‐2* mutant (Supporting Information Figure S4).

### 2.4 Antioxidant treatment during the ARS restores Wt lifespan and INS‐22::Venus secretion in *MSRA‐1* mutants

We then decided to test whether treatment with the antioxidant agent NAC (*N*‐acetyl cysteine) could restore Wt lifespan and insulin secretion levels during the ARS.

**Figure 5:**
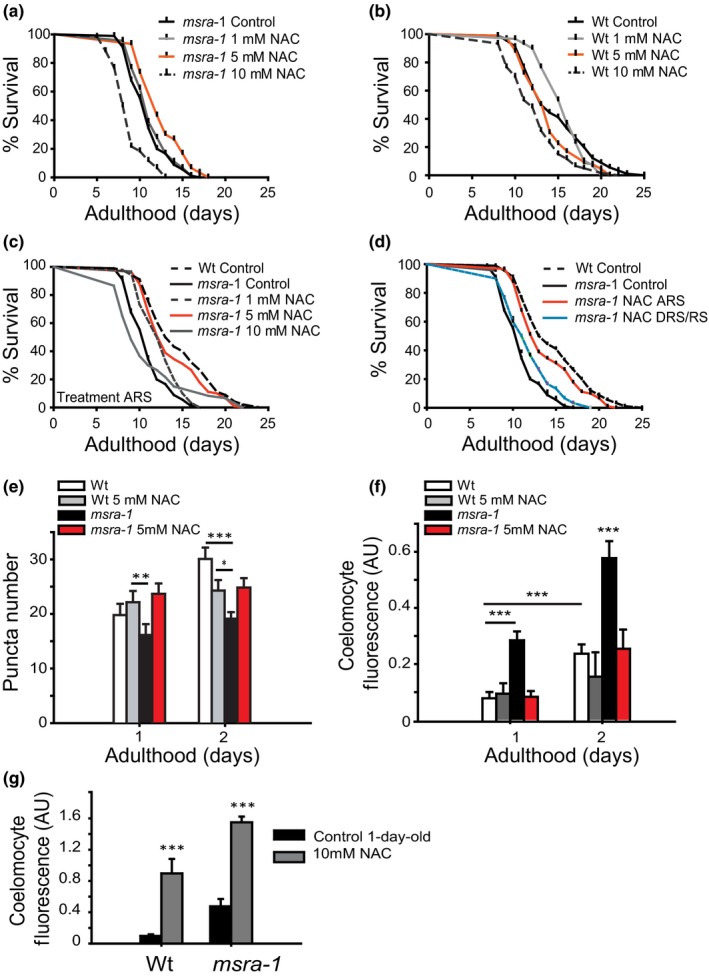
Treatment with the antioxidant NAC exclusively during the ARS restores Wt lifespan and INS‐22::Venus secretion in *msra‐1* mutants. (a, b). Survival curves of worms treated with NAC during adulthood. *p* ≤ 0.0001 for comparison between worms (Wt or *msra‐1* mutants) treated with different NAC concentrations and their corresponding untreated controls. Orange lines correspond to 5 mM treatment. The results correspond to three independent assays, 90 worms per condition. (c) Survival curves of *msra‐1* worms treated with NAC during the ARS. 5 mM NAC during this window of time rescues *msra‐1* mutant lifespan (red line) reaching Wt longevity (black dotted line). (d) Comparison between survival curves of *msra‐1* worms treated with 5 mM NAC during ARS (red line), and from DRS (blue line). *p* ≤ 0.0001 for comparison between *msra‐*1 mutant controls and NAC treated *msra‐1*. The results correspond to three independent assays using at least 90 worms per condition. (e, f). Quantification of INS‐22::Venus released in *msra‐1* worms treated with NAC. (e) INS‐22::Venus puncta number in worms treated with 5 mM NAC. (f) INS‐22::Venus in the coelomocytes of worms treated with 5 mM NAC. (g) INS‐22::Venus in the coelomocytes of Wt and *msra‐1* adult worms treated with 10 mM NAC. Data are means ± *SE* from at least three independent assays. Student's *t* test was used for statistical analysis. ***p < 0.0001. At least 25 animals per treatment and per time point were tested

First, we treated *msra‐1* mutant worms during the ARS with increasing NAC concentrations (Figure 5a). Worms exposed to NAC 5 mM show the Wt lifespan phenotype (orange line). A higher NAC concentration (10 mM) shows a negative effect on longevity. This effect is also observed in treated Wt worms (Figure 5b). Figure 5c shows that 5 mM NAC treatment during the ARS restores longevity of *msra‐1* mutants to near Wt levels (red line). Figure 5d shows the comparison between NAC treatment during ARS (red line) and DRS plus PRS (blue line). Treatment with 5 mM NAC during DRS/PRS (from day 4 of adulthood) shows no effect on the longevity of *msra‐1* mutants. We next evaluated if NAC treatment also affects INS‐22::Venus secretion. Figure 5e,f shows that the treatment of *msra‐1* mutants with 5 mM NAC during the ARS restores INS‐22::Venus secretion to Wt levels, which correlates with the restored longevity phenotype. Figure 5e shows that treatment with 5 mM NAC restores INS‐22::Venus puncta number in the dorsal nerve cord. This is also evident when we analyze the accumulation of released INS‐22::Venus in the coelomocytes (Figure 5f). Figure 5g shows that treatment with 10 mM NAC increases INS‐22::Venus secretion, which correlates with the negative effect this NAC concentration has on longevity.

### 2.5 The locomotor impairments of *msra‐1* mutants can be reversed by treatment with the antioxidant NAC during the active reproductive stage

**Figure 6:**
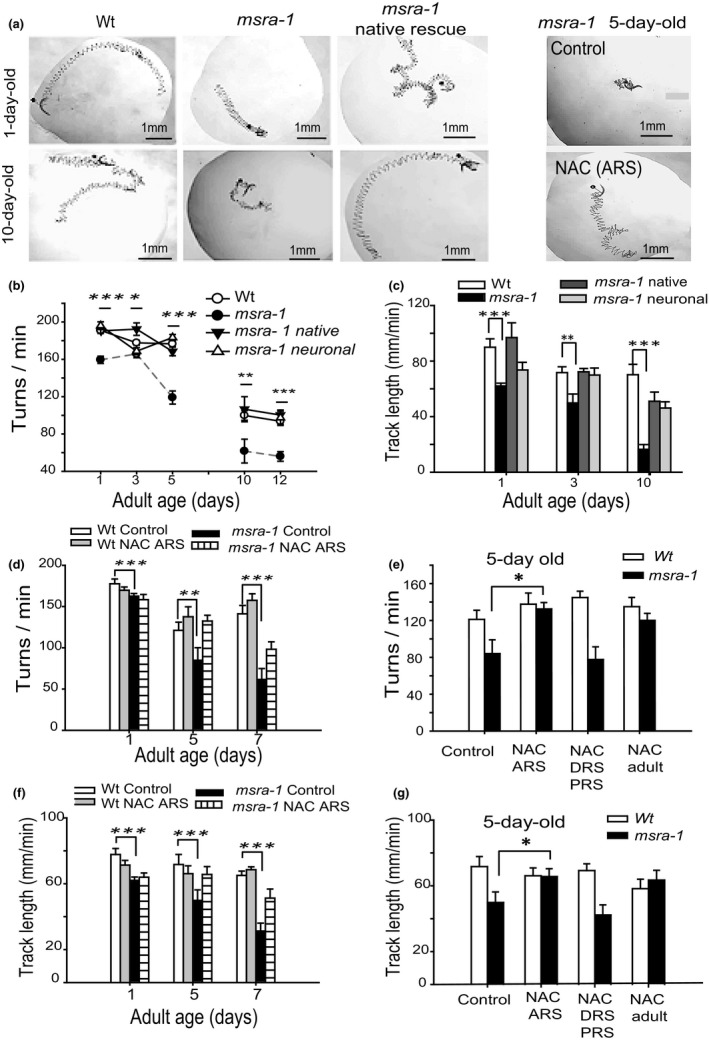
The locomotor impairments of *msra‐1* mutants are reverted by treatment with the antioxidant NAC during the adult ARS. (a) The panels show representative tracks obtained from videos of worms swimming for 1 min. The left panel shows 1‐day‐old and 10‐day‐old worm tracks. The right panel shows 5‐day‐old *msra*‐*1* mutant worm tracks (control and treated with 5 mM NAC during the ARS). (b) The graph shows motility of Wt, *msra‐1* mutant and rescued strains at different adult stages. (c) Track length during swimming. (d) Turns/min of Wt and *msra‐1* mutants treated with 5 mM NAC during the ARS. (e) Comparison of treatment timing with 5 mM NAC on turns/min in 5‐day‐old worms. (f) Track length of Wt and *msra‐1* mutants treated with 5 mM NAC during the ARS. (g) Effect of treatment timing with 5 mM NAC on track length in 5‐day‐old worms. Data are means ± *SE* from at least three independent assays. Student's *t* test was used for statistical analysis to compare Wt and mutants. ***p < 0.0001, ** *p* < 0.001, *p < 0.01. At least 30 animals were tested per strain at each time point

The concept of healthspan has gained importance since it is the period of time that the organism remains healthy and not merely alive (Herndon et al., 2002; Podshivalova, Kerr, & Kenyon, 2017). Therefore, we also wanted to assess the effects of NAC on the aging associated phenotypes of the short‐lived *msra‐1* mutant. These mutants show early locomotor impairments during aging (Minniti et al., 2009). Therefore, we decided to test whether antioxidant treatment during the active reproductive stage can restore *msra‐1* motility to Wt levels. We analyzed swimming capacity through two parameters: turns/min and trajectory length. Figure 6a (left panel) shows representative images of 1‐ and 10‐day‐old worms of different genotypes: Wt, *msra‐1* mutants and rescued *msra‐1* mutants (native expression). Figure 6b shows that *msra‐1* mutants (black circles) move slower than Wt worms (white circles) during the adult stage (turns per minute during swimming). However, at the end of the active reproductive stage (3‐day‐old adults), the mutants’ motility worsens abruptly. This is not the case for the Wt, which maintains its motor abilities at least until day 5. The same is true for the rescued mutants. In fact, the expression of *msra‐1* exclusively in the nervous system is sufficient to rescue motor capacity to Wt levels. In the same way, track length during swimming is significantly reduced in the mutants during all stages and the defects can be rescued when *msra‐1* is expressed in the nervous system (Figure 6c). Additionally, we compared the turn angle during swimming. 1‐day‐old adult *msra*‐1 mutants show turn angle values closer to those corresponding to 10‐day‐old Wt worms (Supporting Information Figure S5b,c).

Next, we treated the mutant worms with the antioxidant NAC only during the DRS/PRS. Figure 6d,f shows that NAC treatment does not affect Wt motility. However, by day 5, NAC improves the motility of the mutants (Figure 6e,g). Figure 6e,g also shows that when NAC treatment is applied in the DRS/PRS stage, there is no improvement in the locomotor capacity of the mutant (neither turns/minute nor track length).

## 3 DISCUSSION

In spite of extensive research on the insulin‐IGF‐1/DAF‐2 pathway and its influence on the determination of aging and longevity during the past two decades, the timing of insulin secretion and its consequences are still unknown. Our results show that there is a temporal pattern of insulin release during the animal´s lifespan. Our experimental strategy allows us to separate insulin expression, in DA and DB motorneurons throughout the animal’s lifespan, from insulin release (Ch'ng et al., 2008). There is experimental evidence showing that the regulation of some insulin peptides, of the many present in *C. elegans,* is also influenced at the expression level (Baugh et al., 2011; Ritter et al., 2013). However, in this study, we focused on the secretion of neuropeptides using INS‐22 as representative peptide to investigate what occurs with some of the insulin peptides released from motorneurons. One of the caveats of our experimental approach is that since insulin peptides are also expressed in other neuronal types and also in non‐neuronal tissues (Pierce et al., 2001; Ritter et al., 2013), their secretion pattern could differ from what we report here. It would be interesting to investigate whether what we describe in this work holds true in the case of insulin peptides secreted from other neuronal types such as sensory neurons. We analyzed secretion of two other non‐insulin neuropeptides and found a similar secretion pattern as we report for INS‐22::Venus, which could indicate that this is a common secretion feature of several neuropeptides. Another caveat is that the secretion of INS‐22::Venus may not be the same as that of the native peptide.

We found that the temporal pattern of INS‐22::Venus secretion from motor neurons goes from nearly non‐detectable levels in L4 larvae to maximum levels in two‐day‐old adult worms. This pattern is observed in the Wt, in the short‐lived mutant *msra‐1* and in long‐lived *daf‐2* mutants. The difference between these strains is not in the pattern of INS‐22::Venus secretion but rather in the levels of secretion. Even though there is evidence that the DAF‐2 insulin receptor regulates aging during the adult stage (Dillin et al., 2002), in this work, we report that the insulin‐IGF‐1/DAF‐2 pathway could be regulated temporally by the release of the insulin ligand during the ARS (or perhaps even just during the first hours of adulthood). This suggests that insulin levels during the *C. elegans* early active reproductive period could determine later metabolic processes that influence aging.

One of the main consequences of the inhibition of the insulin pathway that leads to longer lifespan is the activation of the cell´s antioxidant machinery (Tullet, 2015). It has been reported that not all the antioxidant enzymes and redox compounds upregulated by inhibiting the insulin pathway result in a shorter lifespan when mutated (Doonan et al., 2008; Partridge & Gems, 2002). This evidence suggests that there might be genes that are critical for maintaining redox homeostasis that influence aging. MSRA‐1 is an antioxidant enzyme that when absent decreases longevity in *C. elegans* (Minniti et al., 2009) and other organisms from yeast to mammals (Koc et al., 2004; Moskovitz et al., 2001). Moreover, it was reported that this enzyme favors DAF‐16 nuclear localization in *Drosophila,* which suggests it may have a central role in lifespan determination by regulating the insulin pathway (Chung et al., 2010). Therefore, we investigated if MSRA‐1 could regulate the insulin pathway upstream of DAF‐16 in *C. elegans*. We found that indeed, MSRA‐1 negatively regulates INS‐22::Venus release. Increased INS‐22::Venus release (*msra‐1* mutants) correlates with a shorter lifespan; decreased INS‐22::Venus release (*daf‐2* mutants) correlates with longer lifespan. Our results suggest that neurons are not only a main site of insulin expression (Baugh et al., 2011; Ritter et al., 2013), but also a primary tissue where insulin secretion is tightly regulated. The mechanism that governs the temporal regulation of neuropeptide release is unknown; however, there is *C. elegans* data on a few molecules (*goa‐1, tom‐1*,* unc‐64,* and *unc‐31*) involved in the process of dense core vesicle fusion and neuropeptide secretion that support our evidence that increased insulin release is linked to aging (Ch'ng et al., 2008). In mammals, growth hormone, which plays an important role in the regulation of the insulin/insulin‐like growth factor 1 signaling, also affects lifespan (Bartke, Westbrook, Sun, & Ratajczak, 2013). Moreover, human subjects enriched for familial longevity show decreased secretion of growth hormone (van Heemst et al., 2005).

**Figure 7:**
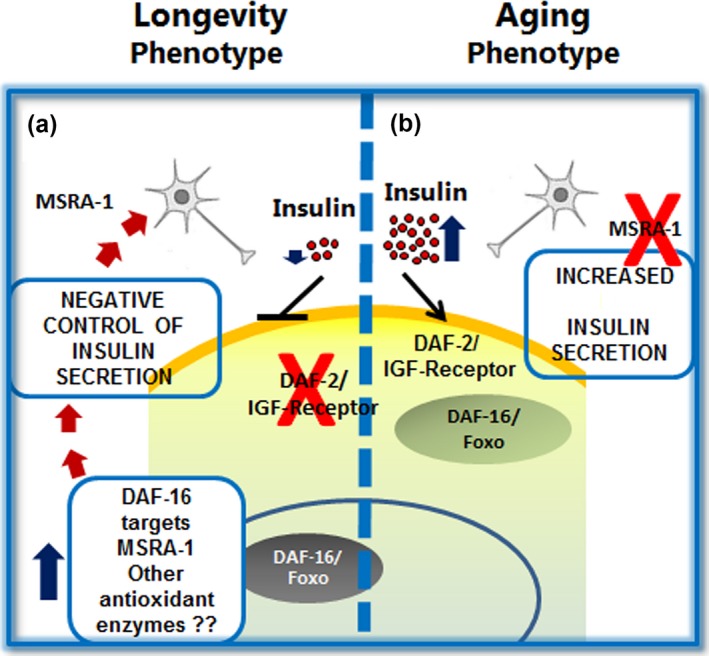
Working model for regulation of insulin release during *Caenorhabditis elegans* young adult stage. Mutations that decrease the activity of the insulin/IGF‐1/DAF‐2 pathway increment longevity by activating the transcription of genes that prolong youthfulness and increase lifespan. Lowering insulin secretion will also shut down this pathway resulting in increased lifespan. Is insulin secretion regulated during the organism lifespan? Here, we show that insulin release is tightly regulated during the *C. elegans* active reproductive stage. Alterations of this regulation result in changes in the aging process. (a) In long‐lived *daf‐2* mutants, the expression of DAF‐16 targets is increased and our results show that INS‐22::Venus secretion levels are barely detectable. One of the DAF‐16 targets that decreases lifespan when mutated is the protein oxidation repair enzyme MSRA‐1. (b) The absence of MSRA‐1 exacerbates INS‐22::Venus release during the active reproductive stage. Expression of MSRA‐1 exclusively in neurons recovers the wild‐type pattern of insulin release and longevity

In this way, during Wt aging, MSRA‐1 may participate in a feedback loop that maintains the insulin pathway downregulated by lowering the availability of insulin ligand/s (see model in the Figure 7). It is known that oxidation on methionines can control the activity of several proteins, such as calmodulin, CaM kinase II, and Aß peptide among many others (Minniti et al., 2015; Oien & Moskovitz, 2008). Therefore, MSRA‐1 could be acting directly on the regulation of proteins involved in dense core vesicle fusion machinery, on proteins that control intracellular calcium levels such as Calmodulin (Lim, Kim, & Levine, 2013), or on proteins that bind Calmodulin (CaM kinase II) (Erickson, He, Grumbach, & Anderson, 2008).

It is possible that other antioxidant molecules that regulate redox homeostasis may also participate in the regulation of insulin secretion.

In order to further test the idea that intracellular redox balance regulates the insulin pathway during adulthood, we exposed *msra‐1* mutants to the antioxidant NAC. As expected, restoration of redox balance through antioxidant treatment of the *msra‐1* mutants only restored health span (measured as locomotor abilities), insulin release, and lifespan to levels similar to Wt when it was administered during the worms´ active reproductive period. After this period, antioxidant treatments are ineffective. It is well known that the organism response to antioxidants displays hormesis, since low levels of these compounds may be beneficial while higher concentrations tend to be detrimental (Schieber & Chandel, 2014). Consistent with this idea, we show that treatment of the *msra‐1* mutants with high concentrations of NAC during the ARS induces higher INS‐22::Venus release and lifespan shortening. It has been shown that redox homeostasis can tightly regulate FoxO activity at various levels including protein synthesis, stability, posttranslational modifications, and subcellular localization (Klotz et al., 2015).

Our evidence supports a mechanism by which redox balance may negatively regulate insulin/neuropeptide secretion from neurons during the ARS (or part of the ARS) in *C. elegans*. In this way, antioxidant enzymes such as MSRA‐1 could participate in a feedback loop that ensures the pathway regulation during the organism´s lifespan. Ultimately, these findings suggest that the insulin‐IGF‐1/DAF‐2 pathway could be regulated only in a narrow window of time during the lifetime of the organism.

## 4 EXPERIMENTAL PROCEDURES

### 4.1 Nematode strains and culture

Nematodes were raised at 20°C (unless otherwise indicated) under standard laboratory conditions (Stiernagle, 2006).

Strains: N2 (Bristol) as the Wt, *msra‐1(tm1421)II,* CB1370 *daf‐2(e1370) III*. Transgenic strains: *nuIs195 [myo‐2::GFP, Punc‐129::ins‐22::Venus] IV;* EG3344 *oxIs180 [Paex‐e::ANF::GFP]*; KP3947 *nuIs183 [unc‐129p::nlp‐21::Venus +myo‐2 p::NLS::GFP]*; GS1912 *arIs37 [Pmyo‐3::ssGFP; dpy‐20(+)] I; dpy‐20(e1282) IV*. ANM47 *msra‐1(tm1421) II,nuIs195 [myo‐2::GFP, Punc‐129::ins‐22::Venus] IV;* ANM49 *daf‐2(e1370) III, nuIs195 [myo‐2::GFP, Punc‐129::ins‐22::Venus] IV;* ANM71* msra‐1(tm1421)II;arIs37 [Pmyo‐3::ssGFP; dpy‐20(+)] I* (this strain may contain* dpy‐20 (e1282));* ANM74 *msra‐1(tm1421)II*; daf‐2(e1370)III; *nuIs195 [myo‐2::GFP, Punc‐129::ins‐22::Venus]IV;* ANM76 *msra‐1(tm1421)II*;* nuIs183 [unc‐129p::nlp‐21::Venus +myo‐2 p::NLS::GFP]*.Transgenic strains were generated by germline transformation (Stiernagle, 2006): ANM56 *msra‐1(tm1421) II,nuIs195 [myo‐2::GFP, Punc‐129::ins‐22::Venus] IV*;* uccEx[ttx3::cherry; prab3::msra‐1];* ANM68 *msra‐1(tm1421)II,nuIs195[myo‐2::GFP, Punc‐129::ins‐22::Venus] IV; uccEx[pmsra‐1::msra‐1;pttx‐3::cherry]*. For RNAi assays we made strains ANM57 *uccEx17[rol‐6(su2006) pmsra‐1::MSRA‐1::GFP] and* ANM75* sid‐1(pk3321) V;oxIs180 [Paex‐e::ANF::GFP]*.

### 4.2 Lifespan analysis

Lifespan analyses were conducted at 20°C as described previously (Kenyon et al., 1993; Minniti et al., 2009) with the following modifications. In all experiments between 25 and 30, L4 hermaphrodites were transferred to plates with OP50 bacteria and containing the chemical 2′‐fluoro‐5‐deoxyuridine 100 μM (FUdR; Sigma) to inhibit the production of progeny (day 0). The worms were scored every 2 days, and they were considered dead when they no longer responded to a gentle prodding with a platinum wire. Worms were transferred to new plates every 2 days. Worms that crawled off the plates during the assay were replaced using the backup plates. All lifespan assays were repeated in at least three independent experiments. Lifespan curves were generated and analyzed with the GraphPad Prism version 5.0. The log‐rank (Mantel‐Cox) test was used for statistical analysis of survival curves.

### 4.3 NAC treatment

Worm populations were exposed to different NAC (*N*‐acetylcysteine, Sigma) concentrations during different periods of time. NAC (diluted in water) was poured onto ready NGM plates before bacteria were seeded to reach the desired final concentration in the agar.

### 4.4 Motility assays

Individual adult animals (between 15 and 25 animals per experiment) were placed on a 30 μl drop of M9 buffer (Stiernagle, 2006). After a 2‐min recovery period, each individual worm was recorded for 1.0 min and the worms´ behavior was analyzed using the wormlab2.0 Software (MBF Bioscience). Turns per minute and track length were automatically examined and quantified as described in (Morales‐Zavala et al., 2017).

### 4.5 Microscopy

Animals were anesthetized in 20 μM NaAzide and mounted on slides. Images were acquired using the same exposure parameters for all experimental conditions, with a 40× objective in an Olympus BX51 microscope (Shinjuku, Tokyo, Japan) equipped with a digital camera Micropublisher 3.3 RTV (JH Technologies, Fremont, CA).

### 4.6 Image fluorescence quantification

Digital quantification of INS22::Venus puncta number in the dorsal nerve cord (anterior or posterior regions nearest to the vulva) and INS22::Venus fluorescence in the posterior coelomocytes was done using the imagej software. Data were estimated as integrated density value using the same threshold parameters for control and treatment situations.

### 4.7 Protein extraction

Collected worms were resuspended in 200 μl lysis buffer in the presence of protease inhibitors (50 mM HEPES pH = 7.5; 6 mM MgCl2; 1 mM EDTA; 75 mM sucrose; 25 mM benzamidine; 1%Triton X‐100) and frozen at 80°C. The samples were sonicated three times on ice for 15 s and centrifuged at 12,000 *g* for 15 min; the supernatants were used for Western blot analysis.

### 4.8 Western analyses

Oxidized methionines in total protein extracts (100 μg per lane) were detected with the rabbit anti‐methionine sulfoxide polyclonal antibody NBP1–06,707 (Novus Biologicals, Littleton, CO). The loading buffer (50 mMTris‐HCl pH6.8; 5% SDS; 10% glycerol; bromophenol blue) lacked DTT and ß‐mercaptoethanol. The proteins were transferred to PVDF membranes. Monoclonal anti‐α‐tubulin antibody (Sigma T5168, Israel) was used as the loading control. Immunoblots for INS22::Venus pro‐peptide were performed in total protein extracts (100 μg per lane) from synchronized worms (2‐day‐old) using the GFP Tag Monoclonal Antibody (11E5) A11121 Invitrogen (USA).

### 4.9 RNAi experiments

These experiments were performed using standard RNAi feeding protocols (Murphy et al., 2003) with the corresponding *Escherichia coli* strain from the Ahringer RNAi feeding library (F43E2.5 clone). The exposure of worms to the *msra‐1* RNAi clone was done from the embryo stage.

### 4.10 Data and statistical analysis

The statistical analyses were performed using the graphpad prism5 software.
