# Supplementary figures and images for: Temporal pattern of neuronal insulin release during Caenorhabditis elegans aging: Role of redox homeostasis
Source: Aging Cell. 2018 Nov 19;18(1):e12855. doi: 10.1111/acel.12855 (PMC6351846; doi:10.1111/acel.12855)

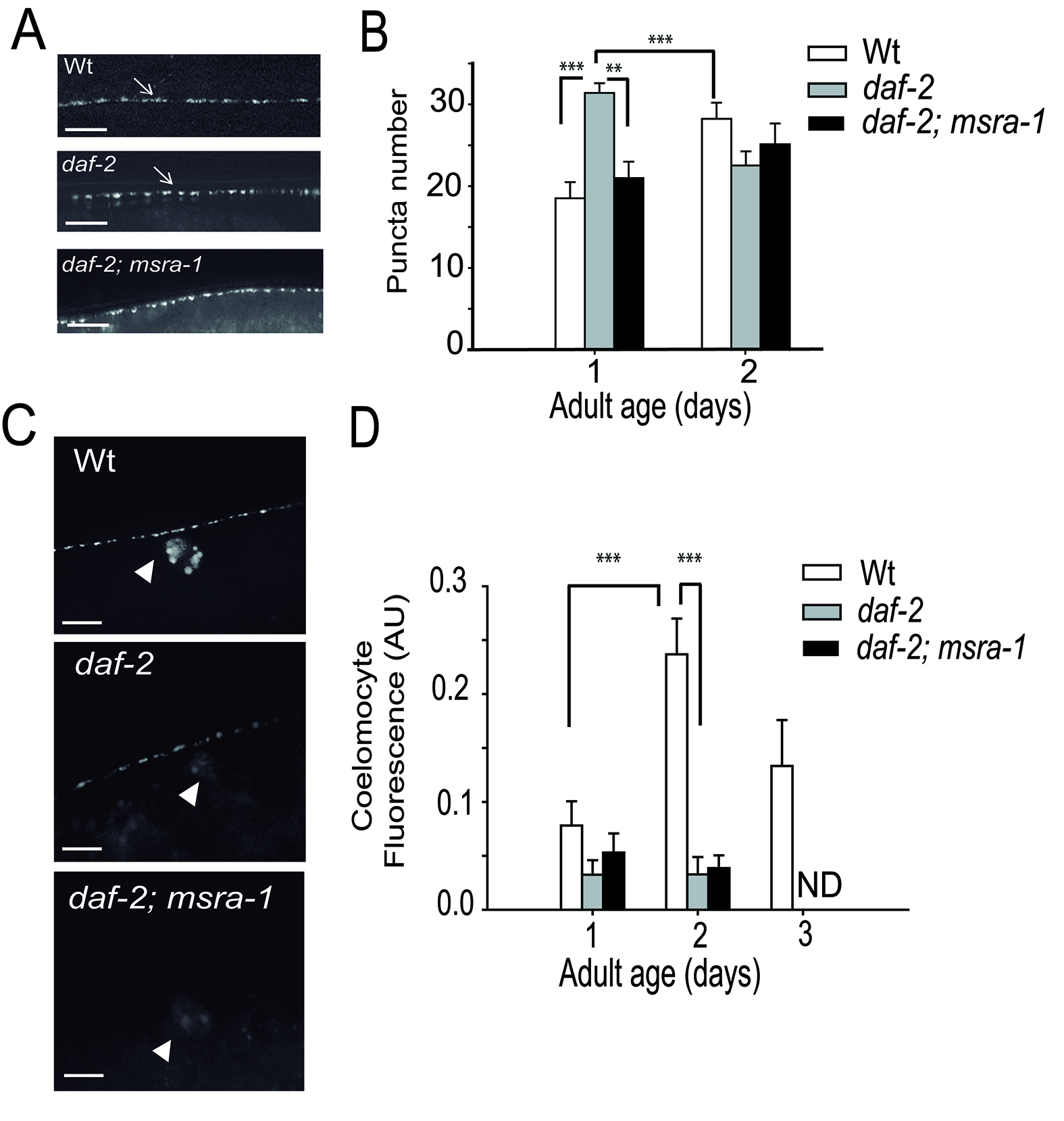

Supplement: Supplementary file 4 [file ACEL-18-e12855-s004.tif]
