# Supplementary material for: Temporal pattern of neuronal insulin release during Caenorhabditis elegans aging: Role of redox homeostasis
Source: Aging Cell. 2018 Nov 19;18(1):e12855. doi: 10.1111/acel.12855 (PMC6351846; doi:10.1111/acel.12855)
Supplement: Supplementary file 6 [file ACEL-18-e12855-s006.docx]

**Legends to Supplemental Figures**

**Supplemental Figure 1**

**ANF::GFP and NLP-21::Venus accumulation into coelomocytes.** A. The graph shows quantification of ANF::GFP (coelomocyte fluorescence) in strain EG3344 from the L4 stage to adulthood day 7. B. The graph shows quantification of ANF::GFP (coelomocyte fluorescence) in strains ANM 75 (fed with control RNAi) and *msra-1* RNAi. C. The graph show the quantification of NLP-21::Venus in strain KP3947 (coelomocyte fluorescence) and *msra-1* mutant worms. Data are means ±SE. Student’s t-test was used for statistical analyses to compare Wt worms of different ages. ***p<0.0001. ND: not detected.

**Supplemental Figure 2**

**5-day-old *msra-1* mutants show increased levels of oxidized methionines.** Quantification of oxidized methionines in total extracts from Wt and *msra-1* worms. A. Representative Western blot analysis of total extracts from 1 and 5-day-old worms. B. The graph shows the densitometric quantification of oxidized methionines in each lane of Western blots from 3 independent assays (all bands were included in the quantification). Data are means ±SE. Student’s t-test was used for statistical analyses to compare Wt and mutants. *p<0.01.

**Supplemental Figure 3**

**A and B. Neuronal expression of INS-22::Venus neuropeptide in Wt, *daf-2* and *msra-1* worms**. **A.** Western blot analysis showing unprocessed INS-22::Venus in DA and DB neurons of 1 day-old worms. This strategy allows us to detect the unprocessed peptide before secretion. The lower bands correspond to GFP/ and processed Venus in the same extracts. Tubulin was used as the loading control. **B.** Densitometric quantification of Western blot analyses. n=3.
**C and D. Accumulation of secreted ssGFP expressed in muscle cells in the coelomocytes of 1-day-old Wt, *daf-2* and *msra-1* mutants.** We used the GS1912 strain that expresses a secreted GFP protein from the *arls37* transgene under the *myo-3* muscle specific promoter to evaluate the endocytic capacity of *daf-2* and *msra-1* mutant coelomocytes compared with the Wt. **C.** Coelomocytes from 2-day-old worms showing accumulation of ssGFP in the different strains. **D.** The graph shows quantification of fluorescence in the coelomocytes from the strains shown in C. Data are means ±SD. n= at least 40 worms per strain.

**Supplemental Figure 4**

**Secretion of INS-22-Venus in Wt, *daf-2* and *daf-2; msra-1* double mutant. A**. Shows representative images of INS-22::Venus in dorsal axons of Wt, *daf-2* and *daf-2*; *msra-1* worms. **B.** The graph shows the quantification of puncta number in all strains analyzed. **C.** Secreted INS-22::Venus in the coelomocytes (arrowheads) in Wt, *daf-2* and *daf-2*; *msra-1* 2-day-old worms. **D.** The graph shows the quantification of fluorescence of INS-22::Venus accumulated in the coelomocytes of Wt, *daf-2* and *daf-2*; *msra-1* worms. ND: not detected. Scale bar, 5 μm. Data are means ± SE. Student’s t-test was used for statistical analysis. ****p*< 0.0001. At least 16 animals were tested at each time point. *daf-2* worms were grown at 16°C until they reached the L4 stage and then shifted to 23°C, therefore the L4 stage could no be analyzed in these experiments.

**Supplemental Figure 5**

**Analyses of *C. elegans* swimming behavior using the WormLab^TM^ software. A.** Shows the trajectory reconstitution of a worm from a 1-minute film (30 frames/sec). Insert: The three points identified in the worm correspond to head, tail and mid-body (center), are automatically detected by the software and used to estimate the turn angle during swimming. **B.** Each graph shows the turn angles of a swimming worm (Wt, *msra-1* and rescued *msra-1*) during 50 sec. Measurements were done at adulthood days 1 and 10. **C.** The table shows the mean turn angle ± SE from 3 different assays. n=30 per strain and age.
